# The intention to disclose medical errors among doctors in a referral hospital in North Malaysia

**DOI:** 10.1186/s12910-016-0161-x

**Published:** 2017-01-23

**Authors:** Arvinder-Singh HS, Abdul Rashid

**Affiliations:** 1 Masters in Health Research (RCSI, Hons), Penang Medical College, Georgetown, Pulau Pinang Malaysia; 2 Clinical Research Centre (Perak), 4th Floor Ambulatory Care Centre (ACC), Raja Permaisuri Bainun Hospital, Jalan Raja Ashman Shah, Ipoh, 30450 Perak Malaysia; 3 Department of Public Health, Penang Medical College, Georgetown, Pulau Pinang Malaysia

## Abstract

**Background:**

In this study, medical errors are defined as unintentional patient harm caused by a doctor’s mistake. This topic, due to limited research, is poorly understood in Malaysia. The objective of this study was to determine the proportion of doctors intending to disclose medical errors, and their attitudes/perception pertaining to medical errors.

**Methods:**

This cross-sectional study was conducted at a tertiary public hospital from July- December 2015 among 276 randomly selected doctors. Data was collected using a standardized and validated self-administered questionnaire intending to measure disclosure and attitudes/perceptions. The scale had four vignettes in total two medical and two surgical. Each vignette consisted of five questions and each question measured the disclosure. Disclosure was categorised as “No Disclosure”, “Partial Disclosure” or “Full Disclosure”. Data was keyed in and analysed using STATA v 13.0.

**Results:**

Only 10.1% (*n* = 28) intended to disclose medical errors. Most respondents felt that they possessed an attitude/perception of adequately disclosing errors to patients. There was a statistically significant difference (*p* < 0.001) when comparing the intention of disclosure with perceived disclosures. Most respondents were in common agreement that disclosing an error would make them less likely to get sued, that minor errors should be reported and that they experienced relief from disclosing errors.

**Conclusion:**

Most doctors in this study would not disclose medical errors although they perceived that the errors were serious and felt responsible for it. Poor disclosure could be due the fear of litigations and improper mechanisms/procedures available for disclosure.

## Background

Medical errors are errors made unintentionally by medical professionals during the care or treatment of patients. These errors can range from a trivial error to a serious life-threatening error. The errors can result in either the patient fully recover, sustain partial recovery with/without disabilities or death. Medical error in this study is defined as patient harm caused by medical doctors only.

Disclosure of a medical error is an attempt by a medical professional to explain to the patient concerning the mistake made medically- whilst or after committing the error [1]. Medical errors are a matter of concern among medical practitioners. As medicine has evolved into vast advancements, the risk of medical errors occurring has increased as well. Along with this evolution in medicine, comes the aspect of empowering patient’s decisions and knowledge concerning their health conditions- including medical errors [2].

Medical errors can be broadly categorized into the following [3]:

1. Diagnostics
  i. Failure to act on results of monitoring/testing
  ii. Error/delay in diagnosis
  iii. Failure to employ indicated tests
  iv. Use of outmoded tests/therapy
2. Treatment
  i. Error in performing an operation/procedure/test
  ii. Error in administering treatment
  iii. Error in dose/method of using a drug
  iv. Avoidable delay in treatment/responding to abnormal tests
  v. Inappropriate care
3. Preventive
  i. Inadequate monitoring/poor follow-up treatment
  ii. Failure to provide prophylactic treatment
4. Others
  i. Failure of communication
  ii. Equipment failure
  iii. Other system failure

To detect a medical error is not entirely easy. Some doctors tend to conceal information from their colleagues let alone staff or patients to avoid medico-legal implications [4, 5]. Medical errors have also been difficult to detect as not only have doctors been known to conceal their mistakes but even at the level of hospital management which may hide medical errors to protect the reputation and business interests of the hospital [6].

The prevalence of disclosure and perception has been reported to range around the world (Europe, Asian countries and in the US) from 39 to 97% [1, 2, 4, 7–12]. The rate of medical error disclosure in Malaysia is relatively unknown- however based on the increasing medical-litigations [13], one can only postulate that the rates are rather lower. There is a dearth in information regarding disclosures in the Asian region as well. A study in Saudi Arabia [14] reported that only 19% of doctors were willing to disclose a medical error to their patients. Among the factors that influenced disclosure included different medical specialities [15, 16] and seniority [17].

As far as actual reporting and perception, Thomas H. Gallagher had looked at the final prevalence of disclosure among doctors and their perception of disclosure. He reported that 65% of doctors would actually disclose medical errors to their patients willingly. However, when the data collected was analysed, the researchers found that the participants were under the perception that 81% were disclosing apparent medical errors and 54% perceived that they were disclosing non-apparent medical errors. In that same study, there was no description of the attitudes of doctors. It was just reported that the attitudes of doctors looking at the medical and surgical scenario were insignificant.

An extensive literature search failed to show published articles on the disclosure or the intention to disclose medical errors by doctors to their patients in Malaysia. Understanding the practise and the factors that influence doctors decision to disclose a medical error is important as it serves as a yardstick for policy makers to see the direction in the practice of autonomy in medicine in the country [18, 19]. It also helps in the empowerment of patients in healthcare and increase the knowledge and understanding of doctors which will subsequently help in the decision to disclosure [20, 21].

### Objectives

The main objective of the study was to determine the proportion of doctors intending to disclose medical errors, their attitudes/perception pertaining medical.

## Methods

### Study design and location

This was a cross sectional study conducted in a tertiary public hospital in north Malaysia from July to December 2015. The hospital chosen for this study was Raja Permaisuri Bainun Hospital of Ipoh. It is a 990-beded tertiary government referral hospital with major speciality personnel available.

### Study sample

The study was conducted among a group of randomly selected doctors from within the hospital. Doctors that were eligible to participate were doctors who were fully registered with the Malaysian Medical Council and on fully employed with the Ministry of Health Malaysia. Doctors who were on long leave/unavailable (medical leave, further studies, short-term attachments etc.) during the time of the study and those who had already participated in this study whilst working in previous departments (to avoid duplication) were excluded.

Using STATA 13 to calculate the sample size and setting the power at 90%, type-I error (alpha) at 0.05 and a difference of 20% (from the studies using by Thomas H. Gallagher et al from and a study in China by Wu et al.- disclosure rates of 65% and 45% respectively) the sample needed was 249 [1, 22, 23]. Using probability proportionate to size method, it was determined that the sample needed was 155 doctors from Medical based specialty and 94 from Surgery based were needed.

### Tools

A self-administered questionnaire was used to collect the information. Using a participant information sheet, the study was explained to the potential respondents and when they agreed, they were requested to sign an informed consent form. They were given 20 minutes to complete the questionnaire. Once they had completed the questionnaire, it was submitted back to the researcher. Doctors who had not returned the forms by the month of December were excluded from the study.

Demographic data of the participants which included age, sex, ethnicity and data in relation to their job including job designation, employment status, average monthly income, highest level of education within the medical field and country, current department working in and the number of years serving within the ministry of health.

The tool that was used to determine the intent to disclose medical errors among doctors was a validated questionnaire developed by Thomas H. Gallagher et al. in the paper titled “Choosing your words carefully” [10]. The validation was done using four vignettes and five item questions. The Cronbach alpha score of the questionnaire was 0.79.

This questionnaire contains four different vignettes (two medical and two surgical based)–

- ○ overdose of insulin (too much insulin given to a patient than the one prescribed- a mistake due to illegible handwriting),
- ○ hyperkalemia (due to a common side-effect from a drug administered and subsequently missed by the doctor),
- ○ retained sponge during a procedure and
- ○ an injury to a bile duct whilst using a new surgical instrument.

The participants answer was analysed and intention of error disclosure was graded into three different categories of “*No disclosure*”, “*Partial disclosure*” or “*Full disclosure*”.

All answer options in the questionnaire were given as statements or one word answers (“Yes”, “No” and Ünsure”). The statement options in the vignettes were given as statements of the choice of words the physicians will choose in an attempt to explain about the error. The words “No Disclosure, “Partial disclosure” and “Full disclosure” were not displayed in the questionnaire, neither was it disclosed to the participants.

The second section measured the attitudes/perception of the participant regarding the medical error. It measured the doctor’s attitude/perception if the situation was a serious error, how responsible the doctor was for the error, the likelihood the doctor would be sued due to the error and how likely the doctor would disclose the error to the patient.

The third section measured the overall review of any medical error encountered by the doctor- disclosing an error would make it less likely that the patient will sue, the likeliness of disclosing an error knowing that the doctor would get sued, the disclosure of near misses that did not harm the patient, informing the patient about minor errors that occurred, disclosing a serious error if the patient is unaware that an error has occurred and the relief of the doctor if he/she decides to disclose a medical error.

Each vignette on disclosure was assessed separately. The measurement of disclosure with the vignettes was done as in the paper by Loren et al. [19]. The doctors that selected partial disclosure were graded as non-disclosure. The answers were finally displayed as “Full disclosure” or “No disclosure”. The reason for these condensations of options was done because the option of “partial disclosure” implied that there was a dishonest element from the respondent (untruthfulness) and did not reveal any error to the patient. Doctors offering to disclose four out of the five questions (80%) for each vignette were deemed to have fully disclosed the error in the vignette. Of the four vignettes- if the doctor was to fully disclose in three vignettes (75%), they were graded as doctors who would overall disclose medical errors.

For the attitudes/perception of disclosure- there were three options the participant could select from- Likely, Unsure and Unlikely. The “Unsure” and “Unlikely” phrases were later combined into one category called “Unlikely”. The reason for this condensation of these two categories was because the researchers were of the opinion that being “unsure” of a disclosure was already bordering the lines of being unethical. If the participant had answered that he/she was likely to disclose the error in at least three of the four vignettes - they were deemed to have a perceived to wanting to disclose an error to the patient. Fully disclosing a medical error was defined as fully disclosing four out of the five questions in the individual vignettes. If a respondent was to fully disclose errors in three out of the four vignettes then they would be classified to fully disclosure.

The overall disclosure attitude/perception was defined as the likely-hood that the doctor is to disclose the error to the patient (last question of attitude/perception assessment for each vignette). If a doctor answered that it was likely for him/her to disclose an error to the patient for three out of four vignettes- he/she is deemed to perceive that a full disclosure is provided.

### Analysis

The data is analysed descriptively. Inferential statistics was done by looking at the factors associated with full disclosure and non-disclosure (partial and no disclosure were condensed further as explained in the “Tool” section). Using chi-square tests, the association of the independent variables (age, job description, place graduated, years of experience and income) with the outcome of full disclosure was measured. The odds ratio was calculated to determine the risk of non-disclosure based on the independent variables.

Chi-square test was also used in inferential statistics to determine the association of the attitudes/perception of doctors disclosing an error and the actual intent of disclosure. This association was quantified into risk using the odds ratio. A bivariate logistic regression analysis was done to predict the type of disclosure based on the different independent variables.

A fitness model test was also performed using the Hosmer-Lemeshow test. This test required a Chi-square association between the disclosure outcomes, followed by the correctly classified percentage for the outcome of disclosure and a Receiver Operator-statistics Curve (ROC).

## Results

There were 449 doctors working at the hospital at the time of the study, 294 were Medical Officers (65.5%) and 155 Specialist (34.5%). Majority of them (*n* = 251, 55.9%) were Medical based, 149 (33.2%) surgical based and 49 (10.9%) were Non-Clinical based (administrative staff). The Non-Clinical staff were included into the Medical based group as they were required to undergo medical rotations before moving into their respective administrative fields.

A total of 276 doctors out of the 397 approached responded giving a response rate of 69.5%. From a total of 121 who were excluded from this study- 70 doctors were on long leave or refused to participate and the remaining 51 did not return the questionnaire.

Table 1 describes the baseline profile of the respondents. The age of the doctors ranged from 24 to 60 years old with the mean age of 33. Most of the respondents were men (*n* = 147, 53.3%) and Indians (*n* = 98, 35.5%) followed by Chinese (*n* = 93, 33.7%) and Malays (*n* = 85, 30.8%). The larger proportion of the respondents were Medical Officers (*n* = 201, 72.8%), and from the Medicine based departments (*n* = 181, 65.6%). The median monthly income of the respondents was RM5,000. Majority (*n* = 144, 52.2%) of the respondents graduated from Malaysian institutions and had only worked within Malaysia (*n* = 253, 91.7%). A very big majority (*n* = 272, 98.5%) of the participants were permanently employed. The mean number of years the respondents worked within the Ministry of Health was 7.9 years.

**Table 1:** Baseline profile of the doctors who participated in the study (*n* = 276)

Variables	n (%; 95% CI)
Age (in years)*	33 (32.42–34.21)
Sex	
Women	129 (46.7; 40.9–52.7)
Men	147 (53.3; 47.3–59.1)
Ethnicity	
Malay	85 (30.8; 25.6–35.5)
Chinese	93 (33.7; 28.3–39.5)
Indian	98 (35.5; 30.1–41.4)
Job Description	
Specialists (inc. consultants)	75 (27.2; 22.2–32.8)
Medical Officers	201 (72.8; 67.2–77.8)
Medical based	95 (34.4; 29.0–40.3)
Surgical based	181 (65.6; 59.7–71.0)
Surgical MO	70 (34.8; 28.5–41.7)
Medical MO	131 (65.2; 58.3–71.5)
Surgical Specialist	25 (33.3; 23.5–44.8)
Medical Specialist	50 (66.7; 55.2–76.5)
Median Monthly Income (RM)**	5,000
Income categorical	
≥ RM 5,000	135 (48.9; 43.0–54.8)
< RM 5,000	141 (51.1; 45.2–57.0)
Place of graduate	
Outside Malaysia	132 (47.8; 42.0–53.8)
Within Malaysia	144 (52.2; 46.2–58.0)
Employment status	
Permanent	272 (98.5; 96.2–99.5)
Contracted	4 (1.5; 0.5–3.8)
Experience of working in countries	
Outside of Malaysia	23 (8.3; 5.6–12.3)
Within Malaysia	253 (91.7; 87.7–94.4)
Years of service in MOH*	7.99 (7.16–8.82)

*Mean (95% Confidence Interval), ** Median

### Overall disclosure of medical errors based on vignettes and the attitude/perception of disclosure

According to the definition set for disclosure, only 28 (10.1%; 95%CI: 7.1 – 14.3) respondents would overall disclose medical errors whereas 248 (89.9%; 85.7–92.9) would not whereas 139 (50.4; 44.4–56.2) perceived that they were disclosing errors to their patients and 137 (49.6; 43.7–55.5) were not.

### Opinion of the participants in relation to the vignettes

Table 2 describes the overall opinions of respondents for each of the vignettes. For the insulin vignette, majority (99.2%) of the physicians felt that the mistake was a serious error and would take the entire responsibility for the insulin overdose mistake (89.1%). Although majority (58.7%) of the respondents felt that they would most likely be sued because of the mistake, most (50.7%) would still disclose the error to the patient. For the hyperkalaemia vignette, 97.2% of doctors felt that it was a serious error. Majority of the respondents (92.0%) felt that they were responsible for the error, and although 60.1% of respondents felt that they would be sued because of the error, 52.5% of them would still disclose the error to the patient.

**Table 2:** Overall opinions of each individual vignettes

Opinion		Vignettes n (%; 95% CI)			
		Insulin overdose	Hyperkalaemia missed	Retained sponge	Bile duct injury
Is this situation is a serious error?	Yes	259 (99.2; 97.0–99.8)	249 (97.2; 94.3–98.7)	253 (96.9;94.0–98.5)	206 (79.5; 74.1–84.0)
	Unsure	2 (0.8; 0.2–3.0)	5 (2.0; 0.8–4.6)	7 (2.7; 1.2–5.5)	39 (15.1; 11.2–20.0)
	No	0*	2 (0.8; 0.1–3.0)***	1 (0.4; 0.1–2.7)*	14 (5.4; 3.2–8.9)**
As a physician, how responsible are you for this error?	Responsible	246 (89.1; 84.8–92.3)	254 (92.0; 88.1–94.8)	251 (91.0; 87.0–93.8)	232 (84.0; 79.2–87.9)
	Unsure	27 (9.8; 6.8–13.9)	20 (7.3; 4.7–11.0)	23 (8.3; 5.6–12.3)	38 (13.8; 10.2–18.4)
	Not responsible	3 (1.1; 0.3–3.3)	2 (0.7; 0.2–2.9)	2 (0.7; 0.2–2.9)	6 (2.2; 1.0–4.8)
How likely do you think that you will be sued because of this error	Likely	162 (58.7; 52.8–64.4)	166 (60.1; 54.2–66.0)	217 (78.6; 73.4–83.1)	125 (45.3; 39.5–51.2)
	Unknown	102 (37.0; 31.4–42.8)	96 (34.8; 29.4–40.6)	50 (18.1; 14.0–23.1)	109 (39.5; 33.9–45.4)
	Unlikely	12 (4.3; 2.5–7.5)	14 (5.1; 3.0–8.4)	9 (3.3; 1.7–6.2)	42 (15.2; 11.4–20.0)
How likely would you be to disclose this error to the patient?	Likely	140 (50.7; 44.8–56.6)	145 (52.5; 46.6–58.4)	192 (69.6; 63.8–74.7)	139 (50.4; 44.4–56.3)
	Unknown	98 (35.5 30.1–44.4)	93 (33.7; 28.3–39.5)	62 (22.4; 17.9–27.8)	89 (32.2; 27.0–38.0)
	Unlikely	38 (13.8; 10.2–18.4)	38 (13.8; 10.2–18.4)	22 (8.0; 5.3–11.8)	48 (17.4; 13.3–22.4)

*15 missing, **17 missing, ***20 missing

Similar to the previous vignettes, the retained sponge was deemed to be a serious error by 96.9% of them, and 91.0% agreed that they were indeed responsible for the error and although 78.6% of them felt that they were likely to be sued for the error, 69.6% would still opt to disclose the error to the patient.

For the bile duct injury (an injury which was caused by a doctor’s negligence and due to unfamiliar use of a new device to perform a cholecystectomy), 79.5% of the participants felt that it was a serious error and most of them (84.0%) felt that the physician in-charge was responsible for the error. Although 45.3% of them felt that they might be sued for the error, 50.4% of them would still disclose the error to the patient.

These disclosures (in all vignettes) however were the measure of attitudes/perception of the respondents that he or she might be disclose which might differ from their choice of disclosing a medical error.

### Attitudes regarding medical errors

Table 3 describes the overall attitudes of the respondents regarding errors. Majority (41.1%) of the participants were in the opinion that they would less likely be sued if they disclosed a serious error. Although majority (35.1%) responded that they were less likely to disclose a serious error if they might get sued, however a substantial number of participants were unsure (32.6%) and disagreed (32.3%). Most of the participants responded that that near misses (44.6%) and minor errors (52.3%) should also be disclosed to the patients. Equal numbers (35.5%) of participants were likely and unlikely to disclose an error to the patient if the patients were unaware of the error. Majority (60.6%) of them were in the opinion that they will feel relief after disclosing an error to the patient.

**Table 3:** Overall attitudes/perceptions of doctors regarding medical errors (*n* = 276)

Attitude/Perceptions assessment questions	Options	Total n (%; 95% CI)
Disclosing a serious error would make it less likely that the patient would sue me	Agree	113 (41.1; 35.4–47.0)
	Unsure	98 (35.6; 30.2–41.5)
	Disagree	64 (23.3; 18.6–28.7)*
I might be less likely to disclose a serious error if I think I might get sued	Agree	97 (35.1; 29.7–41.0)
	Unsure	90 (32.6; 27.3–38.4)
	Disagree	89 (32.3; 27.0–38.0)
Near misses should be disclosed to patients	Agree	123 (44.6;38.8–50.5)
	Unsure	83 (30.1; 25.0–35.8)
	Disagree	70 (25.3; 20.6–30.9)
Minor errors should be disclosed to patients	Agree	144 (52.3;46.4–58.2)
	Unsure	61 (22.2; 17.6–27.5)
	Disagree	70 (25.5;20.6–31.0)*
I might be less likely to disclose a serious error if the patient is unaware that the error happened	Agree	97 (35.5; 30.0–41.4)
	Unsure	79 (29.0; 23.8–34.6)
	Disagree	97 (35.5; 30.0–41.4)***
I experienced relief after disclosing this error to the patient	Agree	166 (60.6;54.6–66.2)
	Unsure	83 (30.3; 25.1–36.0)
	Disagree	25 (9.1; 6.2–13.2)**

*1 Missing, **2 Missing, ***3 Missing

### Inferential statistics

As shown in Table 4, out of the 28 doctors who would disclose medical errors, majority (64.8%) were men. Chinese (42.9%) were more likely to disclose errors followed by Indians (42.9%) and Malays (21.4%) were the least likely. However, the differences in sex and ethnicity were not statistically significant (ethnicity X^2^ = 1.52, *p* = 0.22 and sex X^2^ = 1.66, *p* = 0.44). Participants from the medical based specialities (64.3%) were more likely to disclose errors than the surgical based specialities, this too was not statistically significant (X^2^ = 0.02, *p* = 0.88). The majority of those who disclosed errors were Medical Officers (71.4%) as compared to specialist but again this difference was not statistically significant (X^2^ = 0.03, *p* = 0.86). Similarly the differences in the place where the participant graduated (X^2=^ 0.02, *p* = 0.88) and the working experience was not statistically significant (X^2^ = 0.06, *p* = 1.00).

**Table 4:** Factors associated with the disclosure of errors among the participants

Factors affecting disclosure	Disclose errors	Does not disclose errors	X^2^/ *p* value
	n (%; 95% CI)	n (%; 95% CI)	
	*n* = 28	*n* = 248	
Sex			
Women	10 (35.7; 20.1–55.1)	119 (47.9; 41.8–54.2)	1.52/0.22
Men	18 (64.3; 44.9–79.9)	129 (52.0; 45.8–58.2)	
Ethnicity			
Malay	6 (21.4; 9.8–40.7)	79 (31.9; 26.3–38.0)	1.66/0.44
Chinese	12 (42.9; 25.9–61.7 )	81 (32.7; 27.1–38.8)	
Indian	10 (35.7; 20.1–55.1)	88 (35.5; 29.7–41.7)	
Department			
Surgical based	10 (35.7; 20.1–55.1)	85 (34.3; 28.6–40.4)	0.02/0.88
Medical based	18 (64.3; 45.0–79.9)	163 (65.7; 59.6–71.4)	
Job description			
Specialist	8 (28.6; 14.8–48.1)	67 (27.0; 21.8–32.9)	0.03/0.86
MO	20 (71.4; 51.9–85.3)	181 (73.0; 67.1–78.2)	
Income			
> RM 5000	12 (42.9; 25.9–61.7)	123 (49.6; 43.4–55.8)	0.46/0.50
< RM 5000	16 (57.1; 38.3–74.1)	125 (50.4; 44.2–56.6)	
Place of Graduation			
Outside M’sia	13 (46.4; 28.8–64.9)	119 (48.0; 41.8–54.2)	0.02/0.88
Within M’sia	15 (53.6; 35.1–71.2)	129 (52.0; 45.8–58.2)	
Working experience			
Outside M’sia	2 (7.1; 1.7–25.1)	21 (8.5; 5.6–12.7)	0.06/1.00*
Within M’sia	26 (92.9; 74.9–98.3)	227 (91.5; 87.3–94.4)	

*Fisher exact test used as (>20% of cells had expected count of < 5

### Attitudes/perception of disclosure and actual error disclosure

As shown in Table 5, there is an almost ten-fold odds of a doctor whose attitudes/perception were inclined to disclose an error but not doing so for the vignettes (OR 9.8, CI 2.8–33.3).

**Table 5:** Attitudes/perception of disclosure and actual error disclosure association among doctors responses (*n* = 276)

Factors	Disclosure n (95% CI)		Odds Ratio (95% CI)	(LR *X* ^2^; pseudo R^2^)	*p* value
	No disclosure	Disclosure			
Perception					
Does not disclose error	134 (54.0; 47.8–60.2)	3 (10.7; 3.4–29.0)	1	21.35	<0.001
Discloses error	114 (46.0; 39.8–52.2)	25 (89.3; 71.0–96.6)	9.80 (2.88–33.29)		

### Regression analysis

Regression analysis using age, sex, ethnicity, medical or surgical based departments, specialisation, income, place of graduation and international working experience as possible predictor variables were used in a model to determine associated with the disclosure of errors. A fitness model test was performed. The Hosmer-Lemeshow test showed no significance (*p* = 0.85) as did the Pearson’s chi-square test (*p* = 0.39). The correctly classified percentage was 89.9% and the area under the ROC curve was 64.7%. This suggested that the model is moderately good for analysis. However, none of the variables were found to be statistically significant.

The univariate and multi-factorial binary logistic regression was performed for this study to be compared against the outcome of disclosure/no disclosure. Both analysis had shown that there was no statistically significant difference in the groups of age, sex, ethnicity, medical or surgical based departments, specialisation, income, place of graduation and international working experience with the disclosure outcome.

### Summary of results

Overall, the disclosure by the participants was low, at 10.1%, regardless of their job description. The majority of the respondents would opt for a partial disclosure when disclosing a medical error to their patient. The partial disclosure involves only mentioning what happened to the patient in physiological terms but no mention that it occurred due to a medical negligence. This was however later re-categorised into the “No Disclosure“ for the final analysis.

Most of the respondents were in the opinion that every vignette was a serious medical error and that they were responsible for the medical error that occurred. Most were in agreement that they would not be sued for the error committed if they disclosed an error. The respondents also perceived that they were disclosing enough to their patients. However, there is an almost ten-fold odds of a doctor who perceives he/she will disclose an error not doing so.

All demographic variables which were related to medical error disclosure were analysed and no statistical significance was shown in any as there were far too many non-disclosures than disclosures.

## Discussion

From this study, 10.1% of doctors in Hospital Ipoh will report any sort of medical errors. The prevalence of reporting vary in different countries. A similar study (using the same questionnaire as in the present study) conducted by Thomas H. Gallagher et al. in 2006 [4] to measure the rates of disclosure of doctors in the United States and Canada, reported that 65% of physicians would disclose a medical error. In another study Thomas H. Gallagher et al. conducted in 2006 (in the same region as in 2005), it was reported that the disclosure rate remained the same at 64% (former reported 65%) [8]. In a study conducted by Sherry Espin et al. in 2006 in in the United States and Canada among anaesthesiologist, nurses and surgeons in disclosing errors, reported in that 24% of doctors would fully disclose errors to the patient no matter how small or massive the error was and 90% of physicians mentioned that they felt that the vignette situations were serious errors compared to the 99.2% of respondents in the present study. In this same study, it was reported that only 3% of doctors would not disclose a medical error to the patient even if it bears no consequences to the patient [16]. Similarly, in a literature review done by Kathleen M. Mazor et al. in 2004 [20] intending to evaluate the prevalence of doctors' disclosure, it was reported that only 21% of doctors residing in the United States and Canada had disclosed medical errors that happened to patients. Out of this, 37% admitted that they will disclose an error only if it was to bring a short-term harm to the patient. In another study conducted by Blendon R et al. [24], found that only 35% of patients and their family members in the US felt that they were informed about a medical error that happened to them or their family members. Vincent JL et al. in 1998 [25] who conducted a research on medical error disclosure among physicians in the European Union region, found that 32% of doctors would disclose a medical error and 63% would play down the incident and blame it on something else. In an Italian national study conducted by Domenico Flotta et al. in 2012 [26] reported that 90.2% of doctors felt that they should not conceal medical errors that occur while providing clinical management.

The low rate of disclosing medical errors in this study could be due to the lack of policy in Malaysia that requires physicians to disclose a medical error to their patients. The prevalence of disclosure in the United States and Europe (16) are higher compared to Malaysia probably due to the policy of compulsory patient error reporting by physicians. Another reason could be because of the better patient empowerment mechanisms in place in most Western countries compared to Malaysia. However, error disclosures are generally low due to a number of possible reasons. In a letter sent to the BMJ editor by Hassan Chamsi-Pasha et al. [27], they mentioned that although it was the ethical duty of a doctor to disclose medical errors, doctors are normally advised against it by their lawyers especially if the case has potential medico-legal litigation implications. Other possible reasons for doctors failing to report medical errors could be due to the culture of admitting a mistake is likened to a weak person/profession. Self-reporting is another reason considering that the only person that might be aware of the error could be the physician alone. An avenue for physicians to discuss errors with their own peers and then decide on the best way that the particular mistakes can be avoided may help the physician make the right decision in disclosing an error in the future.

The ethical issue of not informing patients concerning a medical error extends much more beyond the medico-legal litigation, a mistake made by a doctor and not informing the patient may result in a more deadly sequel if the same mistake is repeated by another doctor. A good example would be in a cases of anaphylaxis reaction, mistakes in blood transfusions (wrong blood type administered), wrong drugs being administered and the adverse reaction of a drug (i.e. extra-pyramidal symptoms) etc.[28, 29]. No matter how trivial or serious the medical error that has occurred, it is the solemn duty of the physician to fulfil their ethical obligation by reporting and informing the patient of that error. After all, the basis of early ethics revolved around the trust the physicians had among themselves and with their patients. Even though reporting may cause a rise in medico-legal litigations, it may give more avenues for physicians to improve patient’s clinical management. It might also help reduce the number of fatalities that are occurring especially in countries like the United States (US) where the number of deaths from medical errors were only outnumbered by heart diseases and cancer, but were far higher than people dying from suicide and motor vehicular accidents [30]. It can only be imagined the numbers that are occurring in developing countries which lack policies of reporting medical errors.

### Reasons for choice of disclosure

The fear of litigation prompts most doctors not to disclose medical errors. In the present study, most doctors were in the opinion that they were ready to disclose the errors if they were unlikely to be sued by the patient. A study in Iran by Tagaddosinejad F et al. [8] reported that although 52% of patients were less likely to sue doctors for a medical error if they were informed of the error yet about half of those doctors felt that litigation was still a possibility. In that study majority of the doctors responded that they would disclose a minor error or a near miss and 41.1% might disclose an error to the patient with the hope that the patient might be less likely to sue them.

In a study in Malaysia by Puteri et al. [13] on health medico-legal litigations in the country she reported that the Ministry of Health Malaysia had increased their pay-outs (compensations for court) from RM 1.2 million in 2006 to RM 5.6 million in 2010. From 2005 to 2009 a total of RM 6.6 million (113 cases) had been paid out as compensation through court orders and ex gratia, making it approximately RM58,000 average per case. This statistics could be a reason why most doctors are not disclosing medical errors.

Although lawsuits being a common fear among doctors, there have been other reasons cited for the poor disclosure of medical errors to patients. In a study by Ity Shurtz et al. in 2013 [31] ,who perform a systematic review researching the implications of medical errors towards obstetricians, reported that one of the reason studies have reported low disclosure is the fear of not being able to practice competently anymore.

There are also many reasons why doctors may want to disclose medical errors to their patients. In this study, while collecting data, some consultants shared their experiences regarding knowledge of medical error incidences. Citing an example one senior consultant mentioned that a colleague that was on trial in the court for a medical error had privately admitted to the patient’s family that a medical error was committed. The patient’s family immediately dropped the law suit as they felt that justice had been done and the cause of death to their family member was now known.

In a qualitative study of disclosure expectation of medical errors among doctors in five academic centres in the United States of America reported that the medical error committed was not really the issue but the attitude of disclosure, the manner (mechanism) used to disclose the error and ensuring that the mistake is preventable in the future was the main concern [32]. Suggestions of filling in an admission form and creating focus groups among doctors committing mistakes and doctors who have successfully recovered from the mistakes were among the options suggested as possible means to overcome their trauma of causing a medical error.

Studies have shown that disclosing medical errors provide some level of relief to doctors. Thomas H. Gallagher et al. in 2006 had reported that 60.6% of doctors agreed that they would have some amount of relief if they disclosed errors to patients [4]. This finding is similar to the finding of the present study. In another study conducted by L. Kadjilianin the same year, he reported that 74% of doctors from the United States and Canada would experience relief after disclosure [11]. A previous study conducted by L. Kadjilian et al. in 2006 had reported that a majority of the doctors had answered that they felt some form of comfort or relief after disclosing an error to the patient [6].

### Strengths

This first study of its kind in Malaysia has helped increase the knowledge and understanding of disclosure of medical errors and their associated factors and can be a basis of other studies and reference for future studies in the country.

### Limitations

The limitation in this study is that the investigator presumes that the doctors participating would actually disclose the errors as they have described and not otherwise. This study measures the intent of the doctors but not the actual practice of the doctors.

Many doctors had refused to participate in this survey although being assured that their participation would be anonymous as most were afraid of medical litigation which explains the low response rate of 69.5%. They were under the impression that this study was mooted to expose their shortcomings in disclosure rather than find out the actual standing of doctors on medical error disclosure.

This being a single centre study might not be a reflection of the disclosure rates practiced among doctors in the entire country.

This study did not take into account why do doctors think that mistakes happen. Perhaps future studies should enquire from doctors if they have been involved in committing a medical error before.

## Conclusion

The prevalence of medical errors disclosure among doctors in this study is low. Only 10.1% of doctors chose to disclose medical errors in the vignettes, although many felt that their attitudes/perception were already inclined to adequately disclosing errors to patients. The perception of doctors was that all errors described were of a serious nature and most of them felt responsible for the errors. Doctors felt that they were disclosing adequately to the patient when they were actually not.

### Recommendations

The researchers suggest that doctors should be offered a better support mechanism for error disclosure to patients. Even though many doctors would want to offer disclosure, however the fear of litigation and reputation defamation is an impediment. Proper mechanisms of channelling medical error reporting should be put into place, a mechanism to improve services and not a place to make accusations.

The investigators also suggest patients must be empowered to a right of disclosure if they have been at the receiving end of a medical error. This may make it a compulsory procedure with proper disclosure methods being offered to the patient after an error as done in the western countries of the United States of America and the European Union [1, 25].

The researchers also suggest for this study be conducted at a larger scale (multi-centre) involving district and major referral hospitals to identify the patterns of disclosure among doctors. It will also act as a better representation of medical error disclosure among Malaysian doctors.

Some of the respondents interviewed suggested creating a support group for doctors committing errors as a mean to overcome their trauma of causing a medical error. Perhaps a self-reporting mechanism which has privacy privileges should be set up to help in disclosure as it did for one hospital in Holland [33].

## Abbreviations

ROC: Receiver operator curve
